# Efficacy of Continuous Intravenous Fentanyl for Oral Mucosal Pain in Stevens-Johnson Syndrome: A Case Report

**DOI:** 10.7759/cureus.56735

**Published:** 2024-03-22

**Authors:** Mitsuru Ito, Kazushi Yoshida, Azumi Hidaka, Haruka Mukai, Aki Egawa

**Affiliations:** 1 Pharmacy, The Jikei University Katsushika Medical Center, Tokyo, JPN; 2 Respiratory Medicine, The Jikei University Katsushika Medical Center, Tokyo, JPN

**Keywords:** morphine, stomatitis medicamentosa, oral mucosal lesions, fentanyl, stevens-johnson syndrome (sjs)

## Abstract

The management of oral mucosal pain in Stevens-Johnson syndrome (SJS), known for its severe mucocutaneous reactions, is a significant challenge due to the paucity of effective treatments reported in the literature. This case report aims to help fill this gap by describing the effective use of continuous intravenous fentanyl for the relief of severe oral mucosal pain in a patient with SJS. A patient with postoperative recurrence of cervical cancer developed SJS following chemotherapy. She had severe oral mucosal pain that was not relieved by 12.5 mcg/hour fentanyl transdermal patch, a regular medication. This pain was rated 10/10 on the Numerical Pain Rating Scale (NRS), and the patient had dysphagia and difficulty speaking. On admission, intravenous methylprednisolone (1000 mg/day), oral lip treatment with dexamethasone ointment, and oral rinses with azulene-lidocaine mixture were started. Analgesic treatment consisted of a 12.5 mcg/hour fentanyl transdermal patch of the regular medication and 1000 mg/dose of intravenous acetaminophen twice daily. Due to the inadequate efficacy of the transdermal patch, fentanyl was switched from the transdermal patch to a continuous intravenous fentanyl infusion at 20 mcg/hour on day three of admission. This adjustment significantly reduced pain intensity, which decreased to NRS 5/10 on day six of admission, and the patient was able to drink water and speak. Pain relief preceded clinical improvement of stomatitis. Grade 1 somnolence occurred after the start of intravenous fentanyl, but improved with follow-up. There were no other adverse effects such as respiratory depression. This case highlights the potential of intravenous fentanyl in the treatment of oral mucosal pain associated with SJS, although further studies are needed to confirm these findings and to develop comprehensive pain management protocols.

## Introduction

Stevens-Johnson syndrome (SJS) is a rare acute disease characterized by severe epidermal necrolysis involving both the skin and mucosal surfaces, resulting in significant pain [1]. During the active phase of SJS, patients are at risk of fluid and electrolyte imbalances, nutritional disorders, renal failure, liver damage, and sepsis, requiring multidisciplinary management [1,2]. This is followed by a chronic phase in which almost all patients experience a range of sequelae, but in addition to physical sequelae, psychological post-traumatic stress can also occur [3]. Rapid pain relief is necessary because the severe pain caused by oral mucosal lesions not only hinders recovery by restricting oral intake but also causes psychological sequelae. The United Kingdom (UK) guidelines state that no studies have investigated analgesic regimens in SJS and that patient comfort should be ensured using the World Health Organization principles for analgesic methods [1]. Systemic morphine has been reported to be effective for oral mucosal pain in SJS [4], but the literature on the use of alternative opioids for SJS-related oral mucosal pain is limited. Continuous intravenous (IV) fentanyl infusion is a promising alternative, but its use for oral mucosal pain in SJS patients remains understudied. Compared with morphine, fentanyl has fewer adverse events, including constipation, and is safer in patients with renal impairment [5,6]. Despite these advantages, the literature on IV fentanyl for SJS-specific oral mucosal pain was not found in our search. This case report highlights IV fentanyl as a potential alternative for the management of SJS pain and introduces its use for severe oral mucosal pain as a novel and critical improvement over current therapeutic strategies.

## Case presentation

**Figure 1:**
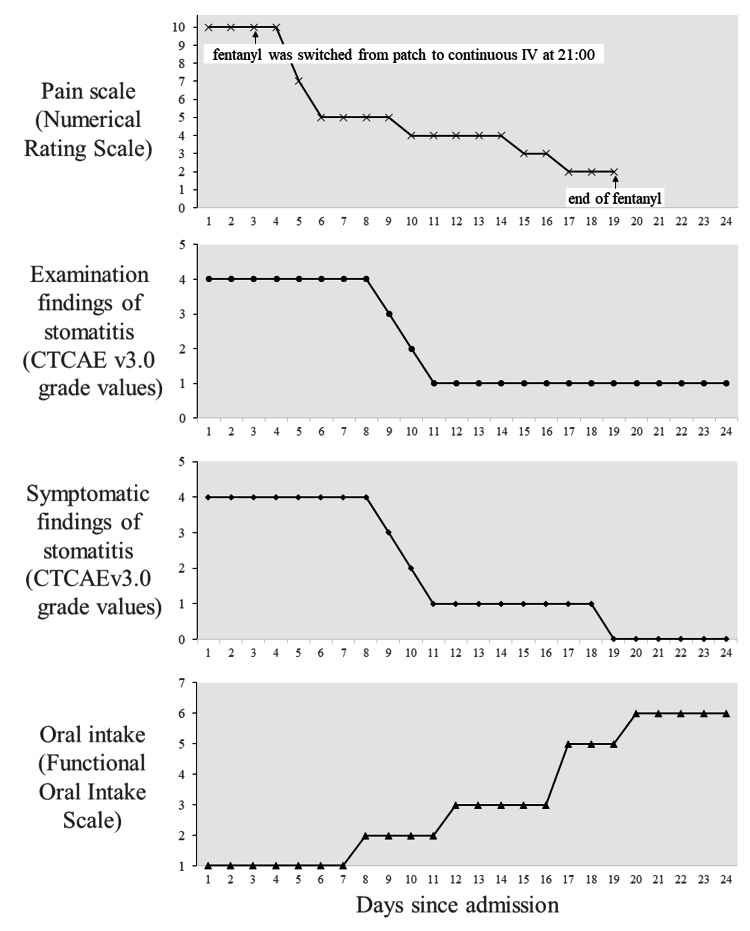
Changes in pain, examination findings of stomatitis, symptomatic findings of stomatitis, and oral intake.

The patient is a woman in her 50s with a postoperative recurrence of cervical cancer. She had a history of asthma and postoperative thyroid cancer; a history of allergy to melon, crustaceans, wheat, and latex; and no history of adverse events. She had back and thigh pain and had been using a fentanyl transdermal patch 12.5 mcg/hour for one month, which effectively controlled the pain. Her other regular medication was oral levothyroxine. She had undergone a radical hysterectomy and bilateral salpingo-oophorectomy seven months previously and one month of postoperative chemoradiotherapy with cisplatin six months previously. Two months ago, a CT scan revealed a mass anterior to the pubic symphysis and on the right side of the pelvis, which was suspected to be disseminated or lymph node metastatic. She had developed a rash and stomatitis after her first round of chemotherapy with carboplatin, paclitaxel, bevacizumab, and pembrolizumab one month earlier. The diagnosis of SJS was confirmed after a detailed examination and pathology. On admission, the patient had erosions and vesicles on her extremities and trunk and multiple oral mucosal erosions with bloody crusts on her lips. Pain was present throughout the body but was most severe in the oral mucosa. Pain was rated 10/10 on the Numerical Pain Rating Scale (NRS), and the patient had dysphagia and difficulty speaking. Initial systemic treatment included three days of IV methylprednisolone (1000 mg/day) starting on day one of admission, followed by IV prednisolone 1 mg/kg tapered over time. At the same time, topical treatments of the mouth and lips with dexamethasone oral ointment and oral rinses with an azulene-lidocaine mixture were started. Analgesic treatment included a continuous fentanyl transdermal patch at 12.5 mcg/hour and 1000 mg doses of IV acetaminophen twice daily. Despite these measures, the patient's oral pain persisted at an NRS level of 10, prompting a change in pain management on day 3 of admission. Fentanyl was changed from 12.5 mcg/h transdermal patch to 20 mcg/hour continuous IV infusion. The dose of the fentanyl transdermal patch is the rate of release, so it is not equivalent to the dose of the IV infusion, but this change was a real increase in dose. Continuous IV infusion was chosen as the switch strategy because of the difficulty of oral medication due to pain swallowing and the need for rapid analgesia. Fentanyl of the same active ingredient was chosen because adverse events were manageable with the usual fentanyl transdermal patch, and the subsequent strategy was to switch to other opioids if ineffective or unmanageable adverse events occurred. Given the increased potential for adverse events associated with higher doses, transcutaneous oxygen saturation using a fingertip pulse oximeter and respiratory rate were measured regularly. This adjustment resulted in a significant reduction in pain intensity, which decreased to an NRS score of 5/10 by day six of admission, allowing the patient to drink water and communicate more effectively. A rescue dose of fentanyl, 20 mcg/dose, was administered once at bedtime as a prophylactic measure on days three and four of admission. Stomatitis was graded as grade 4 (CTCAE v3.0) from day one to day eight of admission, with gradual improvement thereafter (Figure 1).

The only adverse event observed after switching to continuous fentanyl infusion was somnolence, CTCAE v5.0 grade 1, on day six of admission. She was sleepier than usual during the day but was awake. As the somnolence was not associated with respiratory depression or decreased level of consciousness, the same dose of fentanyl was continued under close observation. The somnolence resolved the following day. No other opioid-related adverse events, such as constipation or nausea, were observed. Non-oral pain also improved, although this was not assessed by the NRS. Acetaminophen was discontinued on day six of admission due to the effectiveness of the fentanyl treatment. The continuous fentanyl infusion was maintained at a dose of 20 mcg/hour and was discontinued on day 19 of admission. When the fentanyl infusion was stopped, the fentanyl transdermal patch was not restarted because the back and thigh pain had improved. The patient was discharged on day 24 of admission and outpatient follow-up showed no evidence of sequelae of SJS or long-term adverse events from the medication.

The remarkable reduction in pain achieved in this patient by continuous IV fentanyl infusion highlights its efficacy and leads to a discussion of its wider clinical implications for the treatment of SJS.

This study met ethical standards, and informed consent was obtained from patients.

## Discussion

This case report presents three important clinical findings with implications for the management of severe oral mucosal pain in SJS.

First, the effective relief of oral mucosal pain with continuous IV fentanyl infusion provides an important insight into the management of this debilitating condition. The analgesic effects, which were manifest three days after initiation and preceded observable improvements in stomatitis, underscore the potency and rapid onset of IV fentanyl. Compared with systemic morphine, which Valeyrie-Allanore et al. found to be effective for SJS pain [4], continuous IV fentanyl is an important alternative because it has fewer adverse events and is easier to use in patients with impaired renal function [5,6]. A clear advantage of fentanyl in terms of adverse events is that it causes less opioid-induced bowel dysfunction and does not interfere with nutritional therapy [7]. Hutchinson et al. reported that IV fentanyl caused fewer pruritic adverse events than IV morphine [8]. This may be an advantage when used for the management of SJS. The pharmacological superiority of fentanyl, attributed to its higher mu-opioid receptor affinity, is consistent with the findings of Zhou et al. who reported increased mu-opioid receptor expression in inflamed tissues [9,10]. This suggests that the mechanism of action of fentanyl may be particularly well-suited to conditions characterized by significant inflammation, such as SJS. On the other hand, recent evidence suggests that long-term stimulation of peripheral mu-opioid receptors is involved in the development of opioid tolerance, suggesting the need for cautious use in chronic opioid users [10]. Studies of opioid receptor expression in oral mucositis have been reported by Charbaji et al., but no other reports were found in our search, highlighting the limitations of receptor-based opioid selection and the need for further research [11]. However, reports of the efficacy of IV and transdermal fentanyl for oral mucosal pain in various conditions support the efficacy of μ-opioid receptor agonists [12-16]. Studies using fentanyl for oral mucosal pain are listed in Table 1.

**Table 1:** Comparison of related literature on the use of fentanyl for mucositis. These studies demonstrated the analgesic effects of fentanyl for mucositis pain. The adverse event profile should be interpreted with caution as it may include the effects of the procedure that caused the mucositis. Abbreviations: HSCT, hematopoietic stem cell transplantation; NRS, Numerical Rating Scale; ESCC, esophageal squamous cell carcinoma; NCI CTC, National Cancer Institute Common Toxicity Criteria; NPC, nasopharyngeal carcinoma; SCT, stem cell transplantation; IVF, intravenous fentanyl; TDF, transdermal fentanyl; VAS, visual analogue scale

Reference No	Study Design	Patients	Number of Patients	Gender/Age	Intervention	Dosage and Administration	Outcome Measures	Key Findings	Adverse Event
[12]	Prospective, single-centre, single-arm, open-label	Stomatitis/pharyngo-esophagitis post-HSCT (NRS ≥5 or analgesic request)	15	Female:male 1:3, Age median: 48 (17–65)	IVF	Initiated at 12.5 mcg/hr and adjusted according to pain	NRS score	Mean NRS: Pre-treatment 6.3±1.9, Max dosage 4.9±2.5 (P<0.05).	Nausea 6.7% (discontinuation), Delirium 6.7%, Drowsiness 6.7%
[13]	Prospective, single-centre, single-arm, open-label	Oral mucositis due to chemoradiotherapy in ESCC (NRS ≥4 or NCI CTC grade >1)	46	Female:male: 1: 2.8, Age ≤55 45.7% >55 5,4.3%	TDF	Initiated at 25 mcg/h; adjusted post-24h for mucositis pain to achieve NRS ≤3, with 25 mcg/h increments	NRS score	Median NRS: Pre-treatment 6 (3-9), Day 3: 4.5 (2-9), Day 6: 3 (2-8), Day 9: 2.5 (1-8), Day 11: 2 (0-6), Day 15: 0 (0-4) (all P<0.001).	Nausea/vomiting 15.2%, Dizziness 13.0%, Stomach discomfort 10.9%, Constipation 8.7%
[14]	Prospective, single-centre, single-arm, open-label	Oral mucositis due to chemoradiotherapy in NPC (NRS >5 and NCI CTC grade >1)	78	Female:male 1:3.1, Age median: 41 (31–52)	TDF	Initiated at 25 mcg/h; increased by 25 mcg/h to maintain NRS ≤3 after 24h if no pain control changes	NRS score	Mean NRS: Pre-treatment 7.41±0.96, Day 1: 5.54±0.86, Day 4: 3.27±0.73, Day 7: 2.88±0.62, Day 10: 2.82±0.68 (all P<0.001). Median NRS: 24h post-TDF 5, 72h 3	Nausea/vomiting 10.26%, Dizziness 5.13%, Stomach upset 3.85%, Skin rash 1.28%, Constipation 3.85%
[15]	Prospective, single-centre, single-arm, open-label	Oral mucositis due to chemotherapy (NRS ≥4 or NCI CTC grade >1)	32	Female:male 1.3:1, Age median: 40 (4–76)	TDF	Initiated at 25 mcg/h (adults), 12.5 mcg/h (pediatrics); adjusted post-24h for mucositis pain to achieve NRS ≤3, with 25 mcg/h increments (12.5 mcg/h for pediatrics)	NRS score	Median NRS progression: Pre-treatment 6 (4-9), Day 3: 4 (0-9), Day 5: 2.5 (0-8), Day 7: 2 (0-8), Day 10: 2 (0-6), Day 15: 0 (0-5) (all P<0.001).	Nausea/vomiting 18.8%, Dizziness 15.6%, Stomach discomfort 15.6%, Constipation 6.3%, Itching 6.3%
[16]	Prospective, single-centre, single-arm, open-label	Oral mucositis due to chemotherapy with SCT (autologous/allogeneic) (NCI CTC grade >1)	22	Female:male 1:1, Age median: 32 (17-57)	TDF	Initiated at 25 mcg/h, adjusted to 50 mcg/h or stopped based on daily pain evaluation	from 0 to 10 VAS score	Mean VAS scores: Pre-treatment 6.68, Day 2: 5.17, Day 6: 3.42, Day 10: 2.13 (P< 0.001)	Nausea 31.6%, Dizziness 10.5% Withdrawals: Three patients for severe dizziness, vomiting, body rash after 25 µg/h TTS fentanyl

**Figure 2:**
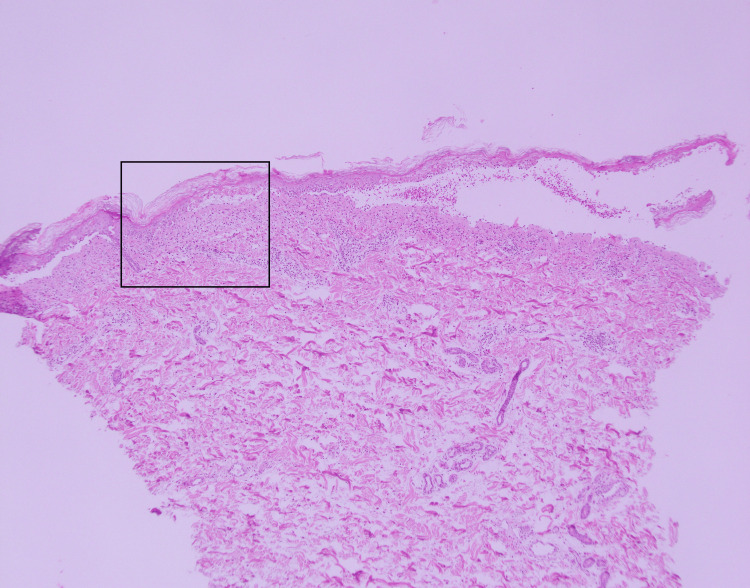
Microscopic image of an edematous, erythematous area of abdominal tissue taken on day one of admission. A magnified image of the black frame is shown in Figure 3.

**Figure 3:**
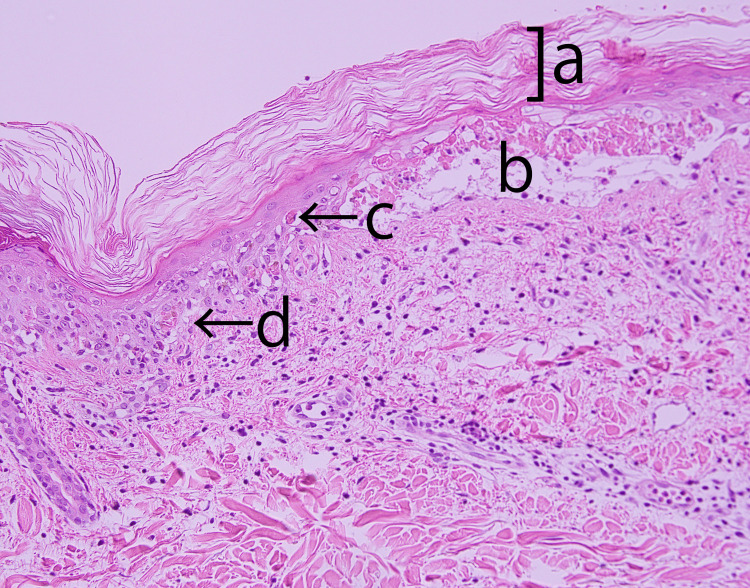
Magnified view of the epidermis and illustration of the lesion. a. Mild hyperkeratosis; b. Subepidermal bullae; c. Necrotic keratinocytes; d. Interface dermatitis

Second, the switch from transdermal to IV administration of fentanyl in our case highlights the limitations of transdermal absorption in SJS patients. Gupta et al. highlighted the critical role of the epidermal stratum corneum in drug absorption [17]. In this patient, pathology revealed hyperkeratosis, suggesting decreased transdermal absorption of fentanyl (Figures 2-3). Subepidermal bullae lesions and other abnormalities have been observed, but the effect of skin lesions on drug delivery is largely unknown, as is the effect of skin degeneration on fentanyl absorption.

On the other hand, oral analgesics may be inappropriate in some cases because SJS is associated with dysphagia and bowel problems [1]. These issues suggest that continuous infusion may provide a more reliable and rapid method of pain control in the management of pain in SJS. IV infusion would be recommended because of the benefits of rapid and consistent pain control and the ease of dose adjustment with the sudden changes in clinical status that can occur in severe SJS. This observation has suggested that continuous IV opioid infusion should be considered early in SJS treatment protocols, especially in cases where rapid pain relief is a priority.

A final important finding is the availability of data on the efficacy and safety of continuous IV fentanyl for severe oral mucosal pain in SJS. The administration of continuous IV fentanyl at a dose of 20 mcg/hour was effective, and the adverse event of somnolence, CTCAE v5.0 grade 1, was observed. This dosing approach, dictated by the patient's severe and persistent oral mucosal pain, requires a careful balance between pain control and minimizing the risk of adverse events such as respiratory depression and somnolence. It should be noted that, in this case, the patient was switched from a fentanyl transdermal patch to a continuous IV infusion, and the adverse event profile may be different than when fentanyl was first initiated. As the adverse event profile of opioid use in SJS is not clear, special attention should be paid to adverse events, especially respiratory depression. In general, opioid-induced somnolence is often transient, but in SJS, metabolic abnormalities and organ damage may progress, requiring continuous monitoring of the level of consciousness and respiratory status. The American Society for Pain Management Nursing (ASPMN) guidelines for monitoring opioid-induced progressive sedation and respiratory depression recommend the use of sedation scales to prevent and detect adverse events when using opioids [18]. The PRODIGY trial identified risk factors for respiratory depression and reported the usefulness of monitoring both ventilation and oxygenation [19,20]. When planning treatment with continuous opioid infusion, these safety measures should be considered and optimized for individual patient conditions.

Our findings highlight a gap in the existing literature on opioid pain management for oral mucositis in SJS and may inform the choice of opioids for pain management.

## Conclusions

Our case report demonstrates the efficacy of continuous IV fentanyl for severe oral mucosal pain in SJS, suggesting a pivotal shift toward more effective pain management strategies to improve patients' quality of life. The introduction of continuous IV fentanyl infusion represents a significant advance by providing a feasible, rapid, and manageable method of pain relief and highlights the need for further research to refine and substantiate these preliminary findings. The integration of this approach into clinical practice holds the promise of improving the care and quality of life of patients suffering from SJS and represents a major step forward in the management of this complex disease. On the other hand, the adverse event of somnolence was observed in the present case and should be carefully considered along with other adverse events such as respiratory depression if used in other cases. The inherent limitations of drawing conclusions from a single case report warrant caution in broad clinical application and underscore the importance of individualized treatment plans.

Given the promising results, further validation through controlled trials is essential to develop evidence-based guidelines, a step made difficult by the rarity of SJS. Despite these challenges, obtaining more case studies for comparison with other analgesics is achievable and beneficial, providing practical insights into alternative pain management strategies and significantly increasing the evidence base for improving treatment methods.
